# Discovering the Influences of Complex Network Effects on Recovering Large Scale Multiagent Systems

**DOI:** 10.1155/2014/407639

**Published:** 2014-04-02

**Authors:** Yang Xu, Pengfei Liu, Xiang Li, Wei Ren

**Affiliations:** School of Computer Science and Engineering, University of Electronic Science and Technology of China, Chengdu, Sichuan 611731, China

## Abstract

Building efficient distributed coordination algorithms is critical for the large scale multiagent system design, and the communication network has been shown as a key factor to influence system performance even under the same coordination protocol. Although many distributed algorithm designs have been proved to be feasible to build their functions in the large scale multiagent systems as claimed, the performances may not be stable if the multiagent networks were organized with different complex network topologies. For example, if the network was recovered from the broken links or disfunction nodes, the network topology might have been shifted. Therefore, their influences on the overall multiagent system performance are unknown. In this paper, we have made an initial effort to find how a standard network recovery policy, MPLS algorithm, may change the network topology of the multiagent system in terms of network congestion. We have established that when the multiagent system is organized as different network topologies according to different complex network attributes, the network shifts in different ways. Those interesting discoveries are helpful to predict how complex network attributes influence on system performance and in turn are useful for new algorithm designs that make a good use of those attributes.

## 1. Introduction

In state of the art of artificial intelligence research, scalable multiagent system applications in complex environments have been popular in domains of military [1], crisis management [2], and business [3]. In those systems, to efficiently coordinate and share information, agents are required to communicate via flexible wireless media, such as mobile ad hoc networks. In those networks, agents may only be able to directly connect with a few of the others and the network topologies may dynamically change according to agents' movements or joint intentions. Moreover, when the system gets bigger, Musolesi et al. have found that the system presents the characteristics of complex networks [4], for example, small world effect discovered by Travers and Milgram [5] and scale free phenomenon discovered by Barabási and Albert [6]. Predicting team performances according to different communication network topologies is interesting and challenging. From our previous research we learned that the same coordination algorithm may lead to huge different performances when the complex network topologies vary [7]. Other researches support our discovery as well. For example, Scerri and Sycara mathematically analyzed how different complex networks may affect the system in information sharing, sensor fusion, and task allocation [8]. Gaston and DesJardins took a bottom-up research on network formation and found that the incomplete complex network structure varies in decentralized adaptation strategies on team performances [9].

Although the effects of complex networks are popular in large multiagent systems, not all distributed algorithms are tested under different complex network topologies in order to estimate how complex network effects change the system performance. As the network recovery is one of the most important network operations to maintain a desired system performance, in this paper, we made an initial effort on finding how the network topology of a multiagent system shifts when agents recover from their communication failures. For example, a UAV may be shot by a hostile missile or robots' connections broken by physical obstacles in a mobile ad hoc network. Although the popular restoration of rerouting mechanism, for example, MPLS recovery algorithm, has been proven to be feasible on network recoveries, the potential shifts on the multiagent network topology may significantly change the system performance in an unpredictable way.

In our simulation, we simulated a series of large scale multiagent systems with different complex network topologies. A popular restoration of rerouting mechanism-MPLS algorithm is implemented to recover link and node failures. The network is evaluated by its diameter, average distance, and cluster [10]. To simulate the physical network, in those experiments, the communication capabilities of each link or node are limited. The experiment results are presented in two major sections: system robustness and influence of network topologies.

In the first section of the experiments, data that flows through the failure agents or links has to be rerouted and may cause congestions on existing links or agents. Based on our discussion about the efficiency of network recovery, the number of newly congested links or nodes (agents) that hurt system performance is investigated. More importantly, the network is in danger of being disconnected from those congestions. By comparing the influence on different network topologies, while there are some differences in different cases, our major discoveries are that a random network is more likely to be congested if more links are broken, while a small world network is the least likely. On the other hand, a scale free network appears to be more vulnerable to node congestions, while a grid network is the most robust.

In the second section, we test how the network topology may be shifted from the network recovery. We have found that most of network topologies are immune to the network recovery when the congestions are not so serious. However, when the number of congested links and nodes is highly increased, the network topologies may have been changed. It is especially the case when the network is organized as a scale free network or a small world network. However, since scale free and small world networks are the most important attributes in a large multiagent system, from our previous research experience, their changes may significantly affect the system performances.

## 2. Related Work

The structure and behavior of complex networks have been attractive in various studies [11]. Inspired by the discoveries on how the rich network structure facilitates effective organizational behavior [12], Gaston and DesJardins illustrated the importance of network topologies in multiagents networks [9]. Y. C. Jiang and J. C. Jiang analyzed the complex network in actor-oriented and actor-structure views to find the relationship between complex networks and multiagent systems [13]. Taylor et al. examined joint actions in the multiagent optimization problem, and the results are surprising because a high number of connections in a complex networked multiagent team can hurt system performance; even communication and computation costs are ignored [14]. Liu et al. proposed an integrated model based on small world network and multiagent system to simulate epidemic spatiotemporal transmission. They found that the small world effect brings better performance than the traditional model, and his discovery has been applied in real geographical multiagent applications [15].

On the other hand, a set of researches show that network operations could influence the performance of the large scale multiagent systems as well. For example, when facing the same network attack, the network vulnerabilities are different if the multiagent systems are organized as various types of complex networks [16]. D'Angelo and Ferretti explained that gossip protocols would change the communication of the complex network and have some impacts on routing efficiency [17]. Gong and Xu analyzed and tested that different parameters on a scale free network can make significant different efficiencies of information delay in multiagent systems [18]. Peschlow et al. [19] described a flexible dynamic partitioning algorithm that rapidly recovers information routing and optimizes the performance with different complex network topologies.

## 3. Modeling the Complex Networks

**Figure 1:**
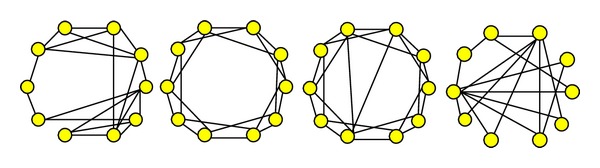
Different complex network topologies: random network, grid network, small world network, and scale free network.

The network topology of a multiagent system is defined as an undirected graph *G* = (*V*, *E*), where *V* = {1,2,…, *N*}, *N* = |*V*|, defines the agent set. *E* denotes the set of links between agents; that is, link (*i*, *j*) ∈ *E* and *i* and *j* are neighbors. *n* : *V* → *E* defines the set of all neighbors of an agent. That is, *n*(*i*) = {*b*, *j*,…, *l*} is the neighbor of agent *i*. *G* could be organized as different network topologies based on the different properties of complex networks. In this paper, we are mainly interested in four of them shown in Figure 1: random network, grid network, small world network, and scale free network. Preliminary studies [20] have found that each topology encodes different fundamental properties, that is, network diameter, average distance between nodes, cluster, and degree distributions.

(i) Degree: the degree of agent *i* is *d*(*i*) = |*n*(*i*)|.
(ii) Average degree: $\bar{d}=\left(1/N\right)\sum_{i\in V}\left|n\left(i\right)\right|$ is the average number of neighbors of all agents, for any complex network $\bar{d}\ll \left|v\right|$.
(iii) Degree distribution: *p*(*k*) = *Pr*[*d* = *k*] defined as a fraction of agents (the number of such agents is *d*) with the degree *k*.
(iv) Distance: distance(*i*, *j*) is defined as the least number of hops to communicate between the agents *i* and *j*. Specifically, distance(*i*, *j*) = 1, if (*i*, *j*) ∈ *E*.
(v) Average distance:
  (1) $$\begin{array}{c} l=\frac{1}{N\left(N+1\right)}\underset{\forall i,j\in V}{\sum }\text{distance}\left(i,j\right) \end{array}$$
  is the average distance between any pairs of agents.
(vi) Network diameter: the diameter of the graph *G* is defined as argmax(*D*), where *D* = {distance(*i*, *j*) | *i*, *j* ∈ *V*}, which is the longest distance between pairs of agents.
(vii) Clustering coefficient: *C*_*i*_ for an agent *i* is given by the proportion of links between the agents within the set of *n*(*i*) that is divided by the number of links between them. The set *e*(*i*) is to record the existing links between these agents in *n*(*i*); then
  (2) $$\begin{array}{c} C_{i}=\frac{2\left|e\left(i\right)\right|}{\left|n\left(i\right)\right|*\left(\left|n\left(i\right)\right|-1\right)}. \end{array}$$
  The clustering coefficient for the network is given by *C* = (1/*N*)∑_*i*=1_^*N*^ *C*_*i*_.

Different complex network topologies can be described according to the properties mentioned above. Erdös and Rényi put forward a classical random network ER model [21]. In this model, a random network follows a Poisson degree distribution. Most nodes in a grid network keep the same degree, which is also called a regular network. Watts and Strogatz put forward the concept of small world network and the WS model [22]. This model presents much shorter average distance than that in a grid network. Moreover, some typical large scale networks such as mobile agents on internet [23] and hyperlinks on web [24] possess certain dynamics—*Matthew effect* [25], a power law distribution: *p*(*k*) ∝ *k* ^−*r*^ (2 < *r* < 3). Some researchers found an interesting formula: argmax(*D*) ∝ lnln*N* [26], that the average distance may decrease as the network grows [27]. This formula precisely reflects the small world effect as well. Then, Barabási and Albert  put forward a scale free network and the BA model [6]. Our simulations are based on those complex network models.

To simulate a physical network, we define the flow of the communication for a link (*i*, *j*) ∈ *E* as *f*(*i*, *j*) and its allowed bandwidth is set as a constant, written as *F*_max⁡_. Therefore, *f*(*i*, *j*) should not overflow to its bandwidth; otherwise, the link will be congested. Please note, if *f*(*i*, *j*) = 0, there is no communication in a physically connected link (*i*, *j*) and it is called a backup link that may be used for future communications. In addition, we define *r*(*i*) as the amount of communication through agent *i*. *r*(*i*) cannot be more than *C*_max⁡_(*i*), the max allowed bandwidth capability through agent *i*; otherwise the agent is congested as well. Moreover, the following properties are defined in our simulations.

- Network connection: *G* is disconnected if ∃*i*, *j* ∈ *V*, distance(*i*, *j*) = *∞*, or no value can be assigned. Otherwise, we say the network *G* is connected.
- Subgraph of a pair of agents 〈*i*, *j*〉: let *G*′(*i*, *j*) be the subgraph of *G*, *G*′(*i*, *j*) = (*V*′, *E*′), where *V*′ is the set of agents on all the shortest paths between the pairs of agents 〈*i*, *j*〉, and *E*′ ⊂ *E* consists of all the links in those shortest paths.
- Transition agents of agent *i* to *j*: let *s*(*i*) be the set to record all the neighbors that can transfer data from agent *i* to *j*, and *s*(*i*)⊂(*n*(*i*)∩*G*′).
- Transition paths of agent *i*: let path(*i*) = {…, 〈*s*, *t*〉,…∣…, *s*, *t*,…∈*n*(*i*)} be the set that records all the pairs of agents that communicate data via agent *i*.

## 4. MPLS-Based Recovery Mechanisms

When agents come to link failures or node failures, the restoration of rerouting mechanisms is usually exploited to maintain the communication between agents. In this paper, we implement a typical network recovery policy, MPLS [28], to restore communication between agents by rerouting mechanisms.

Algorithm 1 briefly describes how a failed link (*i*, *j*) whose data flow is *f*(*i*, *j*) is recovered. We suppose there is a predefined sequence according to agent′ ID that *i*≺*j*. Assume that each agent is able to get the global state of the network, agent *i* can easily find all the alternative shortest paths to *j*, and *w* is one of the transition agents *s*(*i*) (line 1). In line 3, the data flow will be divided evenly into pieces according to the number of shortest paths detected. Each of them will be sent to one of the transition agents and passed through predefined paths (line 3).

Algorithm 2 briefly shows how a failed agent *i* is recovered. The communication through an agent *i* may be composed of several streams from different links. Each stream *p* going through the agent *i* is written as a unique path {…, *u*, *i*, *v*,…}, where *u*≺*v*. The value of data flow going through *p* is written as *f*(*p*). Therefore, to recover node failure, Algorithm 2 first enumerates all the stream path(*i*) (line 1). For each stream *p*, we will find the pair of neighbors of *i* : 〈*u*, *v*〉 in *p*'s path (line 3). Then, if we suppose there has been a link of (*u*, *v*) whose communication amount is *f*(*p*), the stream *p* can be rerouted in a way similar to link failure algorithm (Algorithm 1) (line 4).

## 5. Network Robustness after MPLS Recovery

Although MPLS recovery mechanism can effectively enhance recovery efficiency, network congestion cannot be avoided due to the limited physical communication capacities of the links and agents. In order to detect network congestion including link congestions and node congestions, we designed Algorithm 3 to check existed network congestions around the multiagent system by rerouting data from a link failure (*i*, *j*) or a node failure *j*.

According to Algorithm 3, we can first summarize the link congestion by link (*i*, *j*). It is supposed that the amount of messages conveyed to the link (*i*, *j*) is set as *μ* (line 1). The available capacity *x* for an existing link is calculated as *x* = *F*_max⁡_ − (*μ* + *f*(*i*, *j*)) (line 4). If there is no available capacity, link (*i*, *j*) is congested (lines 5–7). To judge the agents' congestion, we calculate the available capacity *t* for an existing agent *j* as *ε* ← *F*_max⁡_ − *f*(*i*, *j*) and *t* ← *C*_max⁡_ − (*ε* + *r*(*i*)) (lines 6–10). If there is no available capacity, the agent *j* is congested (lines 12–21). The rerouting mechanisms modify the LSP in a failed spot and the length of the shortest path is often more than one agent; therefore, the amount of messages *μ* may be modified (lines 16–19), and the algorithm may be recursively judged many times in terms of Depth First Search (DFS).

In this section, we investigate how network recovering operation may create node or link congestions when *G* is organized as four different network topologies. The system size is *N* = 1000, average degree is $\bar{d}=6$, and maximum allowed link overload is *F*_max⁡_ = 10. The agent having a higher degree usually plays an important role in the network; therefore the communication through the agent is larger. Based on this observation, the capacity of agent *i* follows *C*_max⁡_(*i*) = *λ* × *d*(*i*) × *F*_max⁡_, where 0 < *λ* < 1 is a constant so that its capability is proportional to the degree of the agent. The results are evaluated according to newly congested links, congested agents, probability of network connectivity, and the average distance of the network by varying the ratio of failed links (*Ratio_link*), the ratio of failed nodes (*Ratio_agent*), and the ratio of average communication of each link *f*(*i*, *j*) (*Ratio_flow*). Moreover, when *m* = 0, we assume there are no backup links and all the links are used for communication and, for each (*i*, *j*), *f*(*i*, *j*) > 0. If *m* > 0, additional 15% backup links are presented. The amount of communication for each link is randomly set. The experiment results are based on 100 runs.

### 5.1. Robustness on Link Failure Recovery

**Figure 2:**
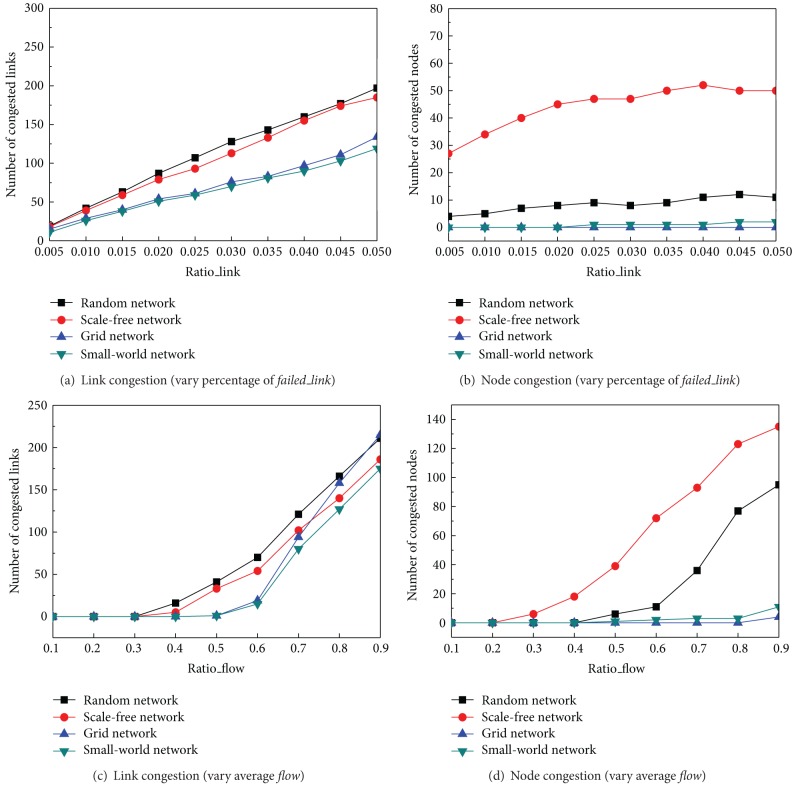
Congestions from link failure recovery by varying* Ratio_link* and* Ratio_flow*.

In this section, we examine the number of congested links and congested nodes when the MPLS algorithm recovered link failures. We fix the average communication of each link *f*(*i*, *j*) as 50% of the *F*_max⁡_ (*Ratio*_*flow* = 0.5). In Figures 2(a) and 2(b), we varied the number of link failures from 0.5% to 5% of all the links. In Figure 2(a), network recovery operation leads to congested links and congested nodes, and their number rapidly increases with the slow increase of* Ratio_link*. By comparing the number of congested links in the same* Ratio_link*, we have found that random network appears to have the largest number of congested links while the small world network appears to have the least number of congested links.

The experimental results in Figure 2(b) show that, except for the grid network, network recovery operation on failed links will lead to node congestions. Apparently, scale free network appears to have the largest number of congested agents in any settings, because of the stability of hub nodes which have large bandwidth (proportion to its degree). The other agents with limited bandwidth are prone to be jammed. Moreover, cluster may contribute to the node congestions as well. For example, the agent *i* whose degree is larger than others' in a scale free network has a larger clustering coefficient *C*_*i*_, and the number of agents retransmitting data would be high. In addition, the cluster of a grid network is the smallest in the four networks, and the node congestions are less likely to be present.

In the next experiment, we fix the failed link as 2% (*Ratio*_*link* = 0.02). Figures 2(c) and 2(d) show that when the average communication* Ratio_flow* of each link *f*(*i*, *j*) increases from 10% to 90% of the *F*_max⁡_, both the congested nodes and links increase no matter what the complex network topologies are. Consistent with the conclusion from Figures 2(a) and 2(b), a random network performs the worst while a small world network performs the best according to congested links. A scale free network appears to have the largest number of congested nodes and random network performs worse as well. The grid network appears to have the least number of congested nodes. It can be explained the same as Figure 2(b).

**Figure 3:**
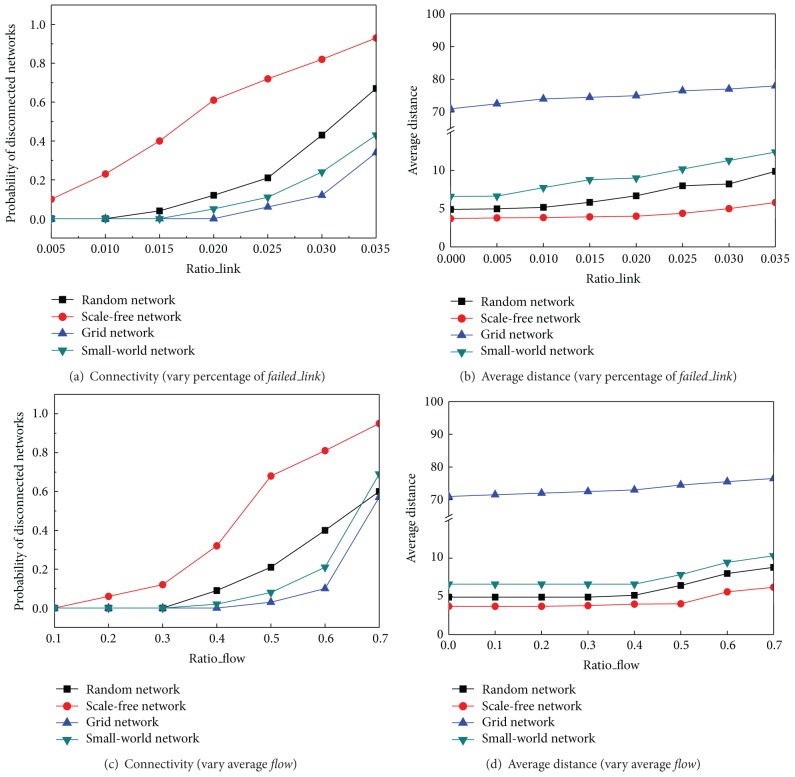
Connectivity and average distance by varying* Ratio_link* and* Ratio_flow*.

**Figure 4:**
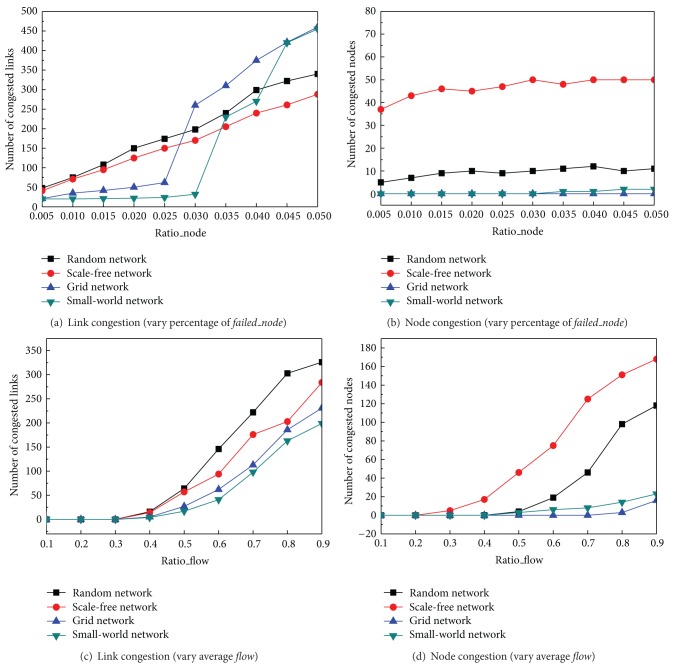
Congestions from node failure recovery by varying* Ratio_node *and* Ratio_flow*.

Figure 4 summarizes how link failure recovery may influence the network performance in two sets of values: the probabilities that the network is broken and the average distance of the network. If network breaks, the system may not work. If the average distance increases, the system's performance decreases because communication flows have to take more hops to the destination. In Figures 3(a) and 3(b), the result shows that when there are more failed links to be fixed (*Ratio*_*flow* = 0.5), the system is in a higher danger to be disconnected. However, a scale free network performs the worst and a grid network performs the best. The reason is that if a hub agent is congested, the network is more likely to be disconnected. On the other hand, agents in a grid network only connect to each other locally; the network is less likely to break down even when more and more links or agents are congested. Figures 3(a) and 3(b) also show that the average distance slowly increases in all network topologies, and, before the network is broken down, the scale free network keeps the shortest distance and reserves the small world effect best. Figures 3(c) and 3(d) illustrate that when the average flow increases (*Ratio*_*link* = 0.02), a similar conclusion can be reached as in Figures 3(a) and 3(b).

### 5.2. Robustness on Node Failure Recovery

In this section, we investigate the network performances after the network recovery policy recovered node failures. In Figures 2(b) and 4(b), we varied the failed agents in the network from 0.5% to 5%, and we set *Ratio*_*flow* = 0.5. We found that the congested links increased quickly while congested agents slowly increased. As we expected, scale free networks made heavy congested nodes. Unlike Figures 2(a) and 4(a), although random and scale free networks have more number of congested links when the failed nodes are sparse, small world and grid networks create about 40% more congested links when failed nodes are more than 3.5% of all the agents.

Figures 4(c) and 4(d) show the results that when we set the failed agents to be fixed as 2% (*Ratio*_*node* = 0.02) and varied the average flow of each link from 10% to 90% of the *F*_max⁡_, both congested agents and congested links are increasing in different complex network topologies. Consistent with the results of link failure recovery, a random network performs the worst according to congested links while a small world network works the best. On the other hand, as we expected, a scale free network always creates more congested nodes while a grid network creates the least.

**Figure 5:**
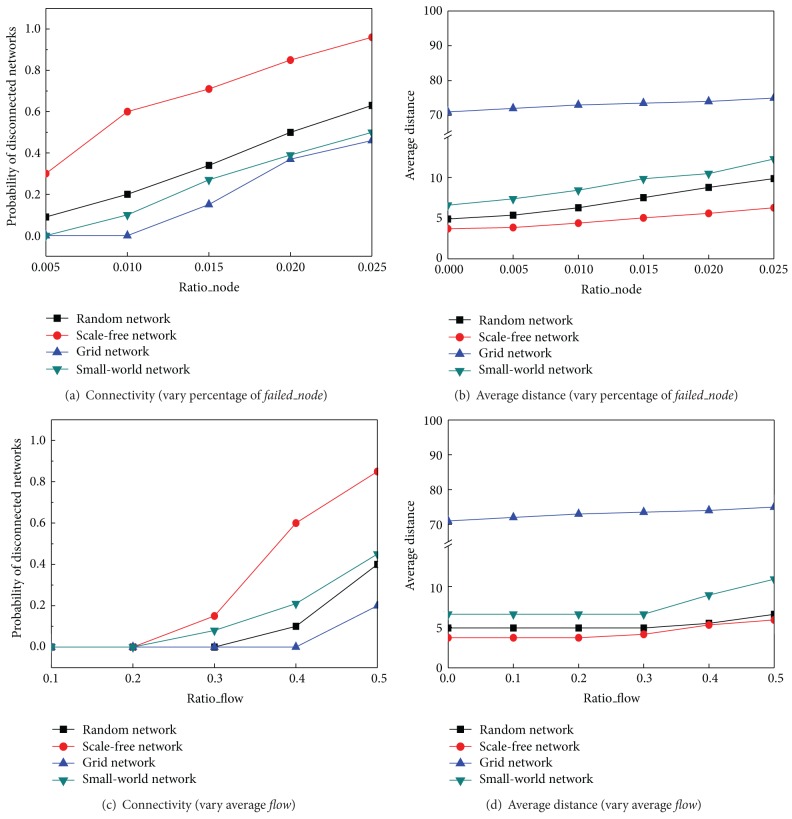
Connectivity and average distance by varying* Ratio*_*node *and* Ratio*_*flow*.

Figure 5 shows when either the rate of failed nodes increases or the average flow rate increases, the probability that the network is broken increases. In Figures 5(a) and 5(b), when there are more failed nodes to be recovered (*Ratio*_*flow* = 0.5), the system is in a higher danger to be disconnected; however, a scale free network performs the worst and a grid network performs the best. The reason is that if any hub agents are congested, the network is much easier to be disconnected. Figures 5(a) and 5(b) also show that the average distance slowly increases in all network topologies, and the scale free network still keeps the shortest average distance as we expected. Figures 5(c) and 5(d) represent that when the average flow increases (*Ratio*_*node* = 0.02), we can make the similar conclusions as Figures 5(a) and 5(b). All the experiments in this section are based on the setting of *m* > 0 (there are 15% backup links), but we could reach the same conclusion when we set *m* = 0.

### 5.3. Data Loss in Network Recovery

As explained, when the multiagent system comes to network failures, although MPLS helps to reroute the data to maintain the system performance, network congestions in the nodes or the links between them will still bring communication loss. In this subsection, we investigate the percentages of data loss when the multiagent system is organized as different complex networks.

**Figure 6:**
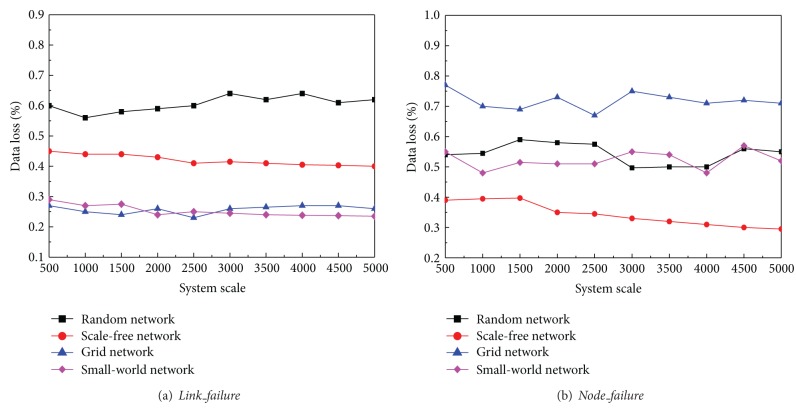
Data loss from network recovery in different network topologies.

In the first experiment, we briefly use the basic setting of Section 5.1 that, in the multiagent system, there are 2% links that are broken and the original average data volume in each link is 50% of *F*_max⁡_. When the system was organized as four different complex network topologies, we measured the percentages of the data loss in different scales. The results are illustrated as in Figure 6(a). We can see that no matter the system size is, the grid network and small world network maintain good performances in link failure recoveries and the data loss rates keep the lowest. Consistent with our analysis in Section 5.1, the scale free network keeps a higher data loss rate and random network performs the worst in link failure recovery where its data loss rate closes to 60%.

In the second experiment, we briefly use the basic setting of Section 5.2, and there are 2% agents that are lost. When the system was organized as four different complex network topologies, Figure 6(b) briefly shows that no matter the system size is, scale free network occupies the stability of hub nodes and always performs the best, and it is especially the case when the network scales up. Consistent with our conclusion in Section 5.2, small world network and random network bring out close performances, while grid network made the worst data loss in node failure recovery. In some cases, the data loss rates are more than 70%.

## 6. Network Shifts on Recovery from Different Topologies

In this section, we verify if network recovery operation would lead to the changes of complex network topologies. Similar experiment settings are kept as Section 5, and both *m* > 0 (consists of 15% backup links) and *m* = 0 are tested. During the experiments, we found that network recovery usually does not lead to distinct shifts of network topologies. However, when the number of congested links and nodes rapidly increases, network connectivity may be destroyed. We set *C*_max⁡_(*i*) = *λ* × *F*_max⁡_, where *λ* > 1 is a constant so that agents' communication volumes are fixed. Our experiments are conducted by varying the parameters* Ratio_link*,* Ratio_node*, and* Ratio_flow* but we always maintain that the network connectivity is not broken (very few results with disconnected networks are excluded). The experiment's results are shown as degree distribution. Each graph represents one type of complex network and consists of three curves with three settings: the original network topology before any failures (*Normal*), the network topology after network recovery (*m* > 0), and the network topology after network recovery without any backup links (*m* = 0).

### 6.1. Link Failure Recovery

In this experiment, we tested how the different network topologies will be changed after link failure recovery. Each network topology will be presented in two different settings with different rate of link failure to be recovered (*Ratio_link*) and different average flow (*Ratio_flow*) which are very likely to break network connectivity.

**Figure 7:**
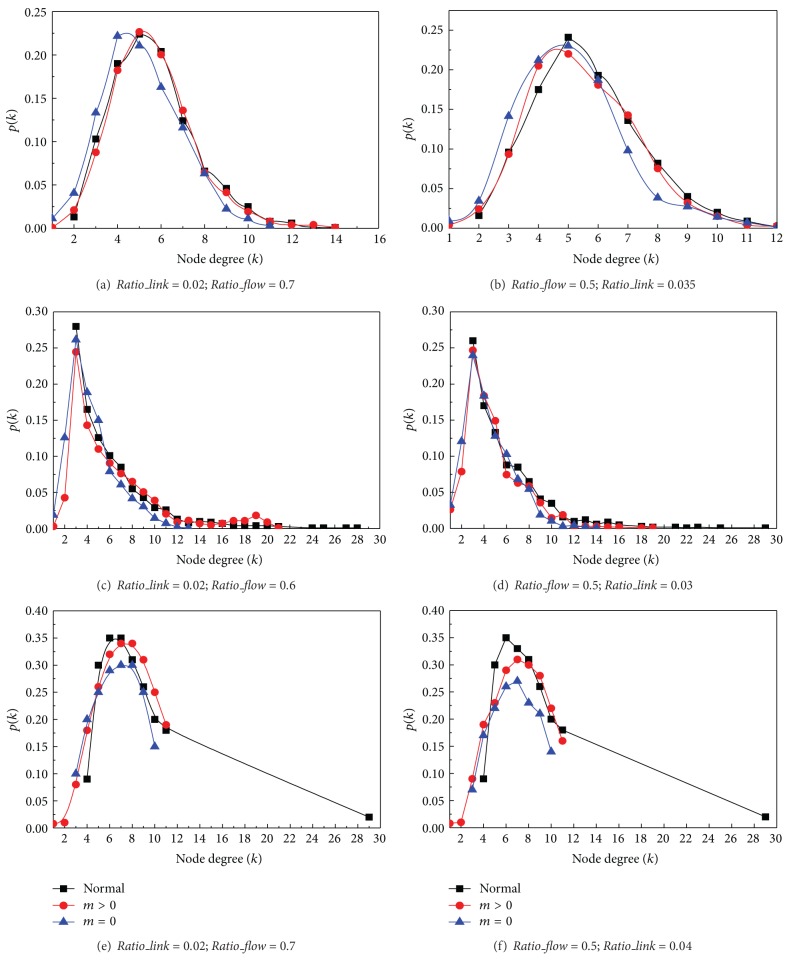
Network shifts from link failure recovery in different network topologies.

Figures 7(a) and 7(b) show how a random network topology shifts on two different settings. Although the random network topology is kept and its distribution still follows a Poisson distribution, the distribution clearly shifts left after the link failure recovered (it is more distinct when *m* = 0). Therefore, its average degree is decreased with link failure recovery.

Figures 7(c) and 7(d) show that the scale free network significantly shifted. In both graphs, the scale free networks are losing their power law distribution and are more and more close to a Poisson distribution as random networks. In the settings of *m* = 0, almost all the high degree agents are lost. The reason is that hub agents are easily congested when much more communication flow transmitted through hub agents. Therefore, the* small world effect* is gradually disappeared.

In Figures 7(e) and 7(f), the original small world network before link recovery presents a generalized binomial distribution [29]. However, the network cannot keep this topology in both graphs. All the agents with higher degrees in a small world network are more likely to be congested, especially in the settings of *m* ≥ 0. Moreover, when the average degree of the networks decreases, the average distance between nodes rapidly increases, and its degree distribution closes to a Poisson distribution after link failure recovery.

### 6.2. Node Failure Recovery

**Figure 8:**
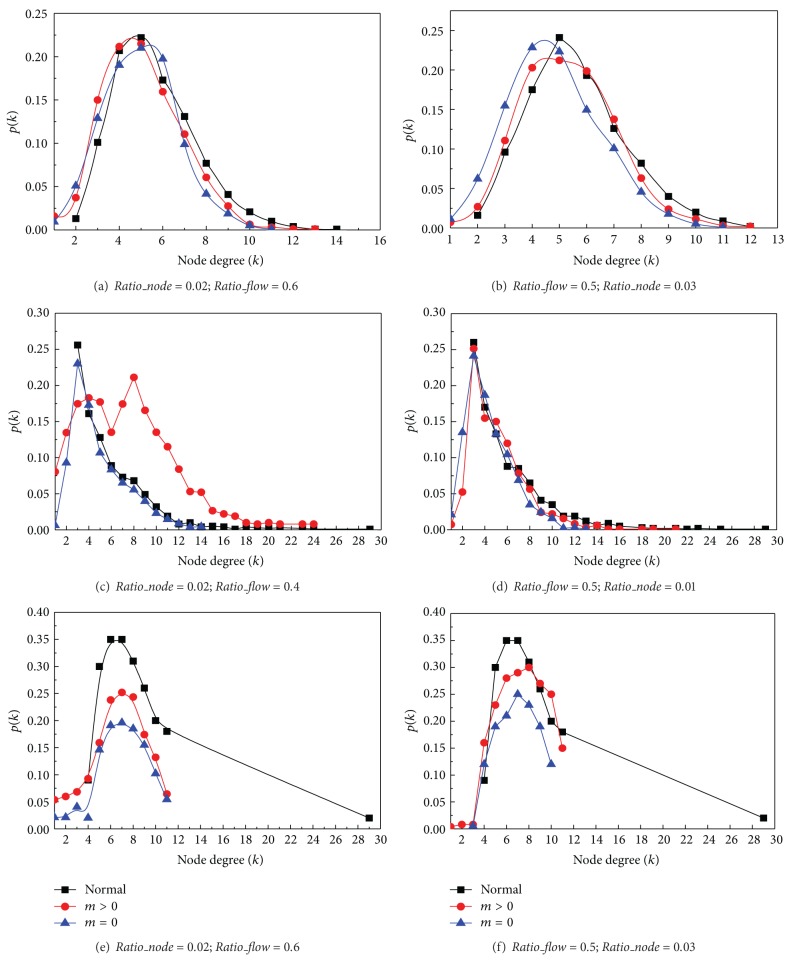
Network shifts from node failure recovery in different network topologies.

Similar to the experiments of link failure recovery in Section 6.1, we vary two parameters of the node failure recoveries:* Ratio_node* and* Ratio_flow*. Similar to link failure recovery schema, networks are very prone to be broken. Figures 8(a) and 8(b) show the changes on the random network. Although the degree distributions slightly change and the average degree decreases, its Poisson distribution remains.

Similar to the conclusion from Figures 7(c) and 7(d), Figures 8(c) and 8(d) show that the scale free networks cannot keep their topologies in both settings and are more and more close to a random network. However, in Figure 8(c), the scale free network has a significant mutation with the setting of *m* > 0. Its degree distribution has been a combination of a Poisson distribution and a power law distribution. We found that the agent *i* whose degree is higher would present higher clustering coefficient *C*_*i*_. Otherwise, low degree agents are loosely connected with each other [30]. Therefore, the scale free network works distinctly between high clustering coefficient region and low clustering coefficient region after node failures are recovered.

Figures 8(e) and 8(f) illustrate that small world networks cannot keep their topologies in node failure recovery. It is similar to the phenomenon in Figures 7(e) and 7(f). In both settings, degree distribution does not follow a generalized binomial distribution any more. Therefore, the average distance between agents may be significantly increased.

In conclusion, the power law distribution in scale free network and the binomial distribution in small network are unstable and can be easily destroyed by network congestions. On the other hand, a random network with Poisson distribution is stable. Moreover, network recovery operation by creating link or node congestions can significantly decrease the average degree of the network. In this section, we excluded the results from the grid network because it only has local connections and the congestions have little influences on its network topology.

## 7. Conclusions and Future Work

Network recovery plays an important role in maintaining the stability of the multiagent system in any application domain. However, its impacts on different complex network organizations are still undiscovered. In this paper, we made our initial efforts on studying those effects. We have found that although the MPLS recovery mechanism can efficiently recover link or node failures by rerouting communication flows via alternative paths, it may bring node or link congestions on those alternative paths. The congestions can significantly change the network topology and system performance. In addition, their effects are different on different complex networks. By conducting extensive experiments, we found that the small world effect and power law phenomenon in a scale free network are not stable in many cases. Based on our interesting discoveries, we may be able to make lots of progresses in near future. The first is to predict the system performance variances according to the changes of the network topology in multiagent coordination domains such as resource allocation, information sharing, and task assignments. Second, we could optimize the recovery algorithm efficiency based on the utilizations of complex network attributes.

## Figures and Tables

**Algorithm 1:**
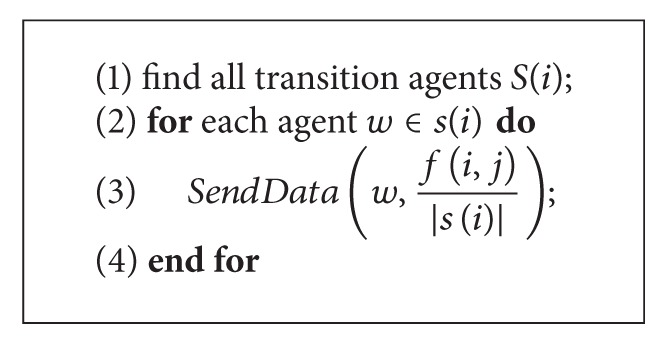
*Li* *nk*_*Recovery*((*i*, *j*), *f*(*i*, *j*)).

**Algorithm 2:**
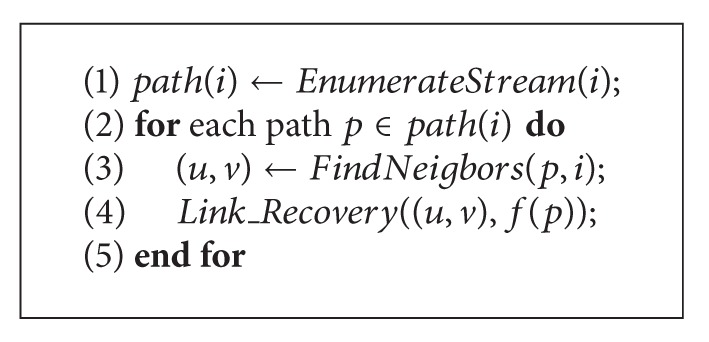
*Ag* *en* *t*_*Recovery*(*i*).

**Algorithm 3:**
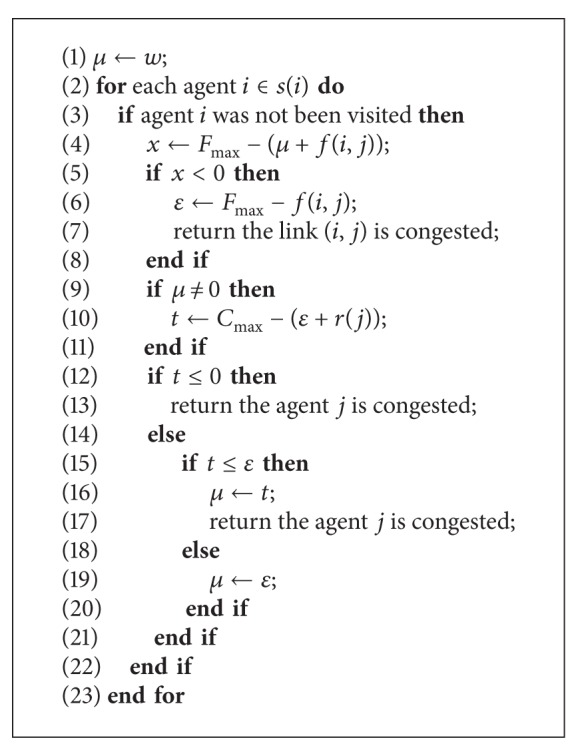
*Congestion*(*i*, *j*).

## References

[1] Wang C, Wang B, Zhang G, Liang Y (2012). Method of formation cooperative air defense decision based on multi-agent system cooperation. *Communications and Information Processing*.

[2] Magarinoa IG, Gutierrezb C (2013). Agent-oriented modeling and development of a system for crisis management. *Expert Systems with Applications*.

[3] Cranefield S, Ranathunga S Embedding agents in business applications using enterprise integration patterns.

[4] Musolesi M, Hailes S, Mascolo C An Ad Hoc mobility model founded on social network theory.

[5] Travers J, Milgram S (1969). An experimental study of the small world problem. *Sociometry*.

[6] Barabási A-L, Albert R (1999). Emergence of scaling in random networks. *Science*.

[7] Xu Y, Lewis M, Sycara K, Scerri P Information sharing in very large teams.

[8] Scerri P, Sycara K (2008). Social networks for effective teams. *Cooperative Networks: Control and Optimization*.

[9] Gaston ME, DesJardins M Agent-organized networks for dynamic team formation.

[10] Albert R, Jeong H, Barabási A-L (2000). Error and attack tolerance of complex networks. *Nature*.

[11] Pagani GA, Aiello M (2013). The power grid as a complex network: a survey. *Physica A: Statistical Mechanics and Its Applications*.

[12] Lin Z, Carley K (1995). DYCORP: a computational framework for examining organizational performance under dynamic conditions. *Journal of Mathematical Sociology*.

[13] Jiang YC, Jiang JC (2013). Understanding social networks from a multi-agent coordination perspective. *IEEE Transactions on Parallel and Distributed Systems*.

[14] Taylor ME, Jain M, n Jin Y, Yooko M, Tambe M When should there be a, “me” in, “team”? Distributed multi-agent optimization under uncertainty.

[15] Liu T, Li X, Liu XP (2010). Integration of small world networks with multi-agent systems for simulating epidemic spatiotemporal transmission. *Chinese Science Bulletin*.

[16] Gu L, Zhang XD, Zhou Q (2011). Consensus and synchronization problems on small-world networks. *Journal of Mathematical Physics*.

[17] D’Angelo G, Ferretti S Simulation of scale-free networks.

[18] Gong XG, Xu J (2013). Research on delay characteristics of information in scale-free networks based on multi-agent simulation. *Procedia Computer Science*.

[19] Peschlow P, Honecker T, Martini P A flexible dynamic partitioning algorithm for optimistic distributed simulation.

[20] Albert R, Barabási A-L (2002). Statistical mechanics of complex networks. *Review of Modern Physics*.

[21] Erdös P, Rényi A (1959). On the evolution of random graphs. *Publications of the Mathematical Institute of the Hungarian Academy of Sciences*.

[22] Watts DJ, Strogatz SH (1998). Collective dynamics of small-world networks. *Nature*.

[23] Bononi L, Bracuto M, D’Angelo G, Donatiello L Exploring the effects of hyper-threading on parallel simulation.

[24] Broder A, Kumar R, Maghoul F (2000). Graph structure in the web. *Computer Networks*.

[25] Merton RK (1968). The Matthew effect in science. *Science*.

[26] Cohen R, Havlin S, Ben-Avraham D (2003). Structural properties of scale-free networks. *Handbook of Graphs and Networks*.

[27] Leskovec J, Kleinberg J, Faloutsos C Graphs over time: densification laws, shrinking diameters and possible explanations.

[28] Sharma V, Hellstrand F Framework for multi-protocol label switching (MPLS)-based recovery.

[29] Mahdi K, Farahat H, Safar M Temporal evolution of social networks in Paltalk.

[30] Yao X, Zhang CS, Chen JW, Li YD (2005). On the formation of degree and cluster-degree correlations in scale-free networks. *Physica A: Statistical Mechanics and its Applications*.

